# AI-Powered Analysis of Eye Tracker Data in Basketball Game

**DOI:** 10.3390/s25113572

**Published:** 2025-06-05

**Authors:** Daniele Lozzi, Ilaria Di Pompeo, Martina Marcaccio, Michela Alemanno, Melanie Krüger, Giuseppe Curcio, Simone Migliore

**Affiliations:** 1 Acquisition, Analysis, Visualization & Imaging Laboratory (A2VI Lab), Department of Life, Health and Environmental Sciences, University of L’Aquila, 67100 L’Aquila, Italy; daniele.lozzi@univaq.it; 2 Department of Biotechnological and Applied Clinical Sciences, University of L’Aquila, 67100 L’Aquila, Italy; ilaria.dipompeo@graduate.univaq.it (I.D.P.); martina.marcaccio@graduate.univaq.it (M.M.); michela.alemanno@graduate.univaq.it (M.A.); simone.migliore@univaq.it (S.M.); 3 Institute of Sports Science, Leibniz University Hannover, 30167 Hannover, Germany; melanie.krueger@sportwiss.uni-hannover.de

**Keywords:** cognitive psychology, eye tracking, Artificial Intelligence, Computer Vision, basketball, sport psychology

## Abstract

This paper outlines a new system for processing of eye-tracking data in basketball live games with two pre-trained Artificial Intelligence (AI) models. The system is designed to process and extract features from data of basketball coaches and referees, recorded with the Pupil Labs Neon Eye Tracker, a device that is specifically optimized for video analysis. The research aims to present a tool useful for understanding their visual attention patterns during the game, what they are attending to, for how long, and their physiological responses, as is evidenced through pupil size changes. AI models are used to monitor events and actions within the game and correlate these with eye-tracking data to provide understanding into referees’ and coaches’ cognitive processes and decision-making. This research contributes to the knowledge of sport psychology and performance analysis by introducing the potential of Artificial Intelligence (AI)-based eye-tracking analysis in sport with wearable technology and light neural networks that are capable of running in real time.

## 1. Introduction

Eye tracking is a technique used to detect an individual’s gaze direction (i.e., where they are looking) and track eye movements in relation to head position. By recording data on the location and duration of visual focus on specific stimuli, this method offers valuable information about visual attention and underlying cognitive mechanisms [1]. In the field of sports, eye tracking is an essential instrument for investigating the visual and cognitive mechanisms underlying athletic performance. Eye tracking technology provides valuable insights into how players perceive and process spatial and temporal information—an essential component for executing accurate and timely decisions during gameplay [2]. By accurately monitoring athletes’ gaze fixations during both training sessions and competitive scenarios, researchers are able to examine perceptual strategies, decision-making processes, and motor planning [3]. This technology is employed across a range of sports—such as basketball, soccer, tennis, and golf—to distinguish the visual behaviors of expert athletes from those of less experienced individuals [4]. Skilled athletes typically demonstrate more efficient visual search behaviors, longer fixation durations, and superior anticipatory skills, all of which contribute to their elevated performance levels [5]. By analyzing these visual patterns, coaches can tailor training programs to help novice players cultivate the perceptual-cognitive abilities required to compete at elite levels [6] Further, eye tracking is not only limited to player performance analysis; it also holds relevance for referees and coaches. For example, referees must rely on rapid and accurate visual assessments to make decisions under pressure. Eye tracking can illuminate how referees allocate their visual attention during critical events, such as three-point shot attempts, and how their expertise influences judgment accuracy [7,8]. Similarly, coaches can use eye tracking data to better understand how players interpret tactical cues, thereby improving instructional strategies and communication [9]. Basketball is a fast-paced, dynamic sport that demands rapid decision-making based on complex visual stimuli. In this sport, across various performance domains—ranging from comparisons between youth and professional athletes during jump shots [10], to expert and semi-elite players executing three-point shots under pressure [5], and even expert versus novice referees [7]—eye-tracking methodologies consistently reveal distinct visual attention patterns that are closely associated with performance efficacy. Notably, expert performers tend to demonstrate extended Quiet Eye (QE) durations and more efficient visual search behaviors, underscoring the critical role of attentional regulation in achieving superior outcomes. Furthermore, eye tracking proves instrumental in evaluating the effectiveness of training protocols and delivering individualized feedback. For instance, Augmented Reality (AR)-based training interventions are shown to enhance both free-throw accuracy and QE duration in novice athletes [11], illustrating the utility of eye-tracking data in refining gaze behaviors and optimizing motor performance. In the field of coaching, eye-tracking research demostrates that instructional strategies involving pointing gestures and guided gaze direction significantly enhance perceptual learning and task performance in novice athletes, whereas such effects diminish with increasing expertise [9,12]. These findings imply a shift in the coach’s role as athlete proficiency develops—from direct, perceptual guidance toward more complex functions such as individualized management, team cohesion, and motivational support. With respect to officiating, referees’ gaze behavior is shown to vary as a function of both court positioning and level of expertise. Expert referees deploy more efficient visual strategies, characterized by rapid and accurate fixation on task-relevant areas. Nevertheless, those positioned further from the ball frequently overlook off-ball events, suggesting limitations in attentional allocation and underscoring the need for training programs that enhance peripheral awareness and improve overall visual coverage of the game environment [7,8]. Moreover, real-time analysis of pupillary responses gains increasing attention as a proxy for cognitive workload and emotional arousal in dynamic environments, including sports officiating, where pupil dilation correlates with stimulus evaluation and decision latency [13]. Additionally, the integration of multi-modal sensor systems, such as Electroencephalography (EEG) and hand-tracking devices (such as Leap Motion Controller^®^, is shown to enhance the ecological validity of perceptual-cognitive assessments by capturing a more holistic picture of human behavior in contextually rich scenarios [14,15,16]. Despite its promise, eye tracking research in basketball faces several limitations. A primary concern is the prevalent use of controlled laboratory environments or simulated game scenarios. While these settings allow for precision and reproducibility, they often fail to replicate the dynamic, unpredictable nature of real-game conditions [10]. Factors such as crowd noise, high-pressure decision-making, and fast-paced interactions are difficult to simulate in lab-based studies, potentially limiting the ecological validity and applicability of the findings. Additionally, much of the current literature [17] focuses on isolated game situations—such as free throws or three-point shots—rather than capturing the fluid, continuous visual behavior of players throughout a full match [18]. This narrow focus restricts our understanding of how athletes adjust their gaze strategies in response to constantly evolving game dynamics. A significant gap in current research is the limited integration of eye tracking with live video analysis. Real-time basketball gameplay introduces considerable complexity, including tracking multiple players, following rapid ball movement, and analyzing intricate player interactions [19]. Data collected in these contexts tend to be more variable and challenging to interpret compared to data from controlled environments [20]. To address this, advanced analytical approaches—such as Machine Learning (ML) and AI—could be leveraged to extract meaningful patterns from high-dimensional data in different fields, from biomedical domain [21,22] to industries [23,24] as well as in sports performance analysis, especially in team sports [25,26]. These technologies allow patterns to be extracted from large datasets. Algorithms of ML, such as Support Vector Regression (SVR) used in [27] for cricket, and those applied to understand decisions in basketball [28], demonstrate the importance of adaptive models for different sports. These technologies hold the potential to uncover subtle and previously undetectable aspects of visual behavior, offering deeper insights into how various actors (i.e., players, referees, coaches) perceive and respond to the game in real time [13]. Within the AI framework, the Deep Learning (DL) emerges for its ability to identify nonlinear patterns and automatically extract features with few preprocessing steps. Automatic analysis technologies, applied to competition videos, make it possible to identify key events (goals, fouls) and track players’ movements [29,30,31]. Algorithms for motion recognition are effective in tracking and classifying activities in sports such as basketball and volleyball, and also find application in injury prevention through accurate assessment of postures and movements [32]. The analysis of complex movement data, including spatiotemporal data and wearable sensor data, offers opportunities to optimize athletic preparation and strategies [33]. These systems provide detailed information on position and movement, allowing analysis of collective actions and informed decisions [34,35,36]. Consequently, an interdisciplinary and data-driven approach, with an increasing role of Computer Vision (CV) [37,38], is crucial in today’s sports landscape. Integration of AI into movement analysis improves understanding of the game, provides tools for injury prevention, and personalizes training [39]. Despite progress, few studies analyze eye movements and pupillometry using CV in ecological contexts (i.e., real matches) [40]. To address this shortcoming, the current study presents a CV system dedicated to the analysis of eye-tracking data in team sports, focused on basketball sport, but extensible to any other sport played within the same conditions (e.g., clearly recognizable game clothes).

## 2. Materials and Methods

### 2.1. Data Acquisition

**Figure 1:**
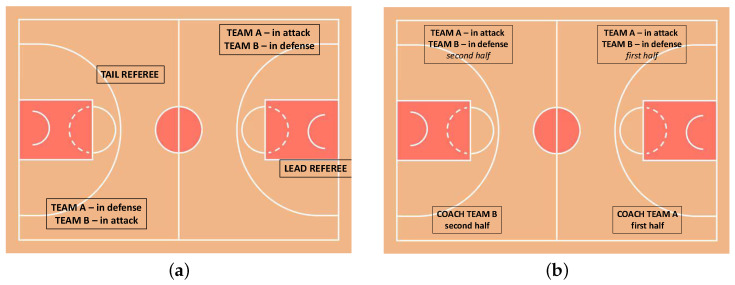
Referee and Coach Observation Conditions. Panel (**a**) illustrates the observational positions of the Lead Referee and Tail Referee on the basketball court, detailing which team’s offensive or defensive play each referee focuses on. Panel (**b**) depicts the game scenarios under which Coach Team A (first half of the game) and Coach Team B (second half of the game) were observes, specifying the quarters and the offensive/defensive roles of each team.

The data are collected during two basketball games with different game situations and different environments in which the two opposing teams wears white-light-(Team A) and blue-dark-(Team B) uniforms. We collect data from four different referees, two for each match: one positioned as the trail referee and the other as the lead referee. The referees’ field positions are illustrated in Figure 1. Specifically, the lead referee observes Team A while attacking and Team B while defending, whereas the trail referee observes Team A while defending and Team B while attacking. Regarding the coaches, data are recorded from four different coaches, two per match. As shown in Figure 1, during the first half, data are recorded from the coach of Team A, with Team A in attack and Team B defending in their own half. In the second half, data are recorded from the coach of Team B, with Team A still in attack and Team B continuing to defend in their own half. The game situations and the individuals involved are illustrated in Figure 1: the panel (a) shows situations in which the referee wears the device, and the panel (b) shows the situations in which the coach wears the device.

### 2.2. Device

The Pupil Lab Neon^®^ eye-tracker device is used in this study for data acquisition (https://pupil-labs.com/products/neon) (accessed on 1 June 2025). This device is an eye-tracking instrument, with a weight of less than 50 gr that uses a neural network for tracking eye movements, with an accuracy of 1.8 degrees uncalibrated and 1.3 degrees with offset correction, thanks to two 192 × 192 pixel InfraRed (IR) cameras at 200 Hz. Furthermore, it integrates a front camera for recording the Point of View (POV) of the user from 1600 × 1200 pixels at 30 Hz with a wide field of view, Inertial Measurement Unit (IMU) sensors and two microphones, with a horizontal angle of 103° and a vertical angle of 77°, and a diagonal Field of View (FOV) of 128°.

### 2.3. Data

Eye tracking features such as gaze, fixations, saccades and pupil diameter are automatically calculated by the device software. Specifically, the system identifies them using algorithms optimized for dynamic contexts and wearable eye trackers, as described by the manufacturer on its official website (https://docs.pupil-labs.com/neon/data-collection/data-streams/) (accessed on 1 June 2025).

Pupil diameter data are also provided by the system for each frame. These derived metrics form the basis for the subsequent analysis presented. The CSV files given as output by Pupil Lab Neon^®^ device provide a detailed recording of each eye-tracking session: (1) the world_timestamps.csv file serves as a temporal reference, synchronizing all other data with the frames of the scene video; (2) the events.csv file records the salient events of the session, such as the start and end of recording or any annotations added by the user, allowing to contextualize the collected data; (3) the gaze.csv file contains the estimates of the gaze position, expressed in 2D coordinates, together with a confidence measure for each estimate; (4) the fixations.csv and (5) saccades.csv files provide processed information on fixations and saccades, fundamental eye movements to understand visual attention; (6) 3d_eye_states.csv offers a deeper look, recording the three-dimensional state of the eye; (7) the blinks.csv file tracks blinks, while (7) imu.csv records the inertial data from the integrated IMU, capturing head movements during the session. Please note that in this study, data are recorded during real basketball matches to build the system and not to analyze the ocular behavior. Future studies will increase the dataset and will focus on the results of the acquired data.

### 2.4. Neural Networks

This study employs two types of Neural Networks. A concise explanation of their functioning and selection for this paper is provided below.

**You Only Look Once neural network v. 8N (YOLOv8N)** [41]: Convolutional Neural Network (CNN) architecture optimized for efficiency and accuracy. It has three primary components of a YOLOv8N model: the backbone, neck, and head. The backbone, responsible for feature extraction, the neck, which facilitates feature fusion that employs a Path Aggregation Network (PAN) structure, enabling the model to effectively integrate features from different scales and the head, tasked with object detection, utilizes a decoupled head approach, separating the classification and regression tasks. This design choice contributes to improved performance and efficiency. This network is designed and pretrained for object-detection.

**Simple and Efficient Design for Semantic Segmentation with Transformers (SegFormer)** [42]: The transformer architecture for semantic segmentation features a hierarchical encoder with both standard and specialized Transformer layers. It starts with overlapping patch merging layers to reduce feature map resolution and capture multi-scale context. These lead into Transformer encoder blocks with self-attention for long-range dependencies and feed-forward networks for non-linearity. SegFormer uses a lightweight all-MLP decoder to aggregate encoder features, producing the segmentation map without complex upsampling, enhancing efficiency. This architecture is designed and trained for semantic segmentation, and in particular clothes for this pretrained version.

### 2.5. Processing Pipeline

**Figure 2:**
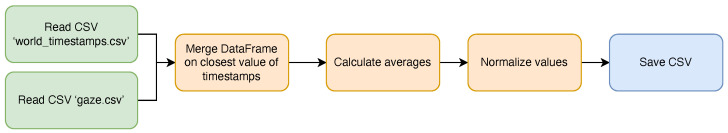
Alignment of two time-series.

**Figure 3:**
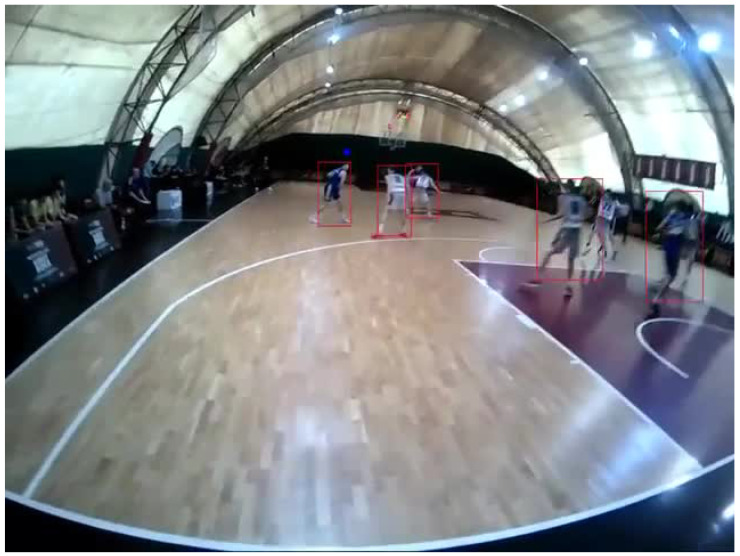
A frame of the video after object detection performed by YOLOv8N: the red boxes represent object detected and the cyan dot is the gaze of the user, in this case the referee.

The preprocessing step includes a human assessment of the video quality for each acquired video. Each of them, length from ∼40 s to ∼4 min, and the events of each scene (attack, defense, etc.) are labeled by expert human raters of basketball sport. The system is written in Python 3.10 with PyTorch [43], Numpy [44], Matplotlib [45] and scikit-learn libraries [46]. The first step of the data processing pipeline, presented in Figure 2, allows the merging of data from the different internal and external cameras. For that, CSV files are imported that contain the video scene timestamps, gaze coordinates, three-dimensional eye states, and event markers. Next, the temporal alignment of the datasets is performed by associating the records in separate tables on the basis of the temporal proximity of the timestamps. This procedure ensures the integration of the different data modes, allowing a holistic analysis of the ocular behavior in relation to the visual context and the recorded events, sampled at the lowest external camera sampling rate (30 Hz). Then, a normalization of the gaze coordinates is performed, mapping them in a normalized space between 0 and 1, in order to make the data comparable. Subsequently, the video from external camera is processed frame by frame, where YOLOv8N detects and tracks objects, as shown in Figure 3.

The process of identifying an object, in this case a player, and the subsequent verification that the user’s gaze falls within its “bounding box” effectively defines a dynamic Area of Interest (AOI). Unlike static AOIs, which are predefined in laboratory studies, our approach allows the analysis to be adapted to the salient elements that are constantly changing in an ecological environment such as a basketball game.

**Figure 4:**
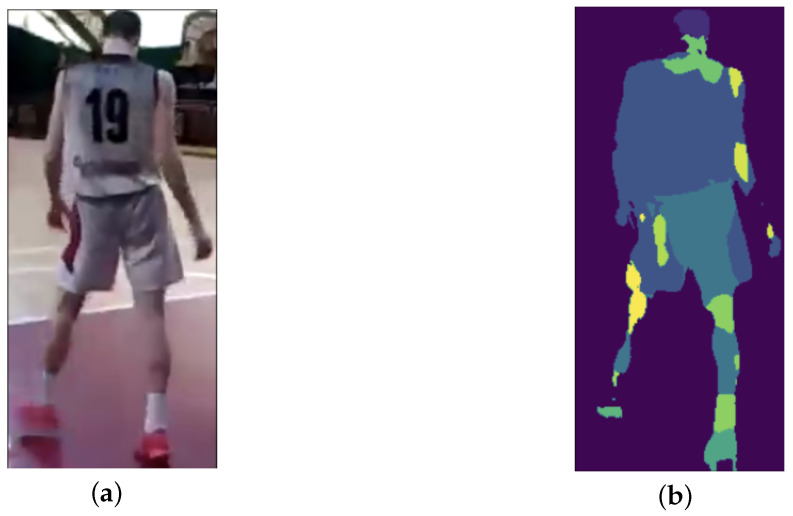
CNNs results of (**a**) YOLOv8N and (**b**) SegFormer. The YOLOv8N (**a**) detect the object and give as output the pixels coordinates of it, and the SegFormer (**b**) segment the clothes. The different colors in (**b**) respresent different clothes segmented by SegFormer neural network.

Frames are saved as images, with bounding boxes and gaze points superimposed. One example result of this step is shown in Figure 4a. The script uses the gaze data to classify when the gaze point is within a bounding box.

Once the dynamic AOI (the observes player) is identified, it crops the detected object and applies semantic segmentation via the SegFormer neural network [42] to isolate the upper clothing (in this case the player’s clothes). This semantic segmentation is shown in Figure 4b. In addition, the script also handles missing frames and errors, ensuring a robust analysis of the video.

**Figure 5:**
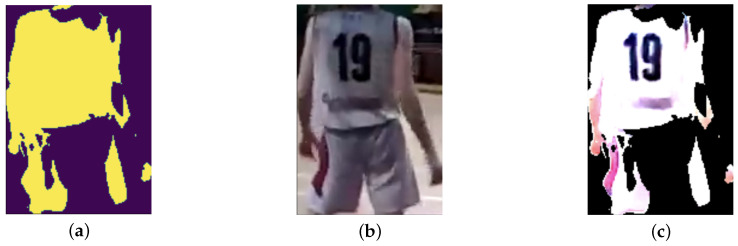
Visual representation of the image processing steps to isolate clothing for color analysis: (**a**) Binary mask generated by SegFormer’s semantic segmentation, identifying clothing pixels. (**b**) Original image cropped to the bounding box detected by YOLOv8N. (**c**) Isolated clothing area, resulting from the element-wise multiplication of the binary mask (**a**) and the cropped image (**b**). The image in (**c**) is scaled using the MinMaxScaler function [46] solely for enhanced human visualization of color differences.

**Figure 6:**
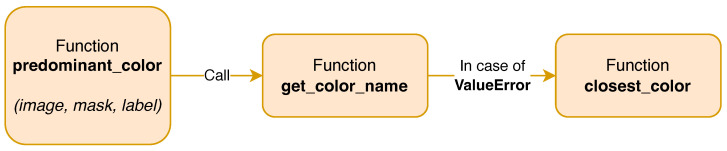
Color detection methodology.

**Figure 7:**
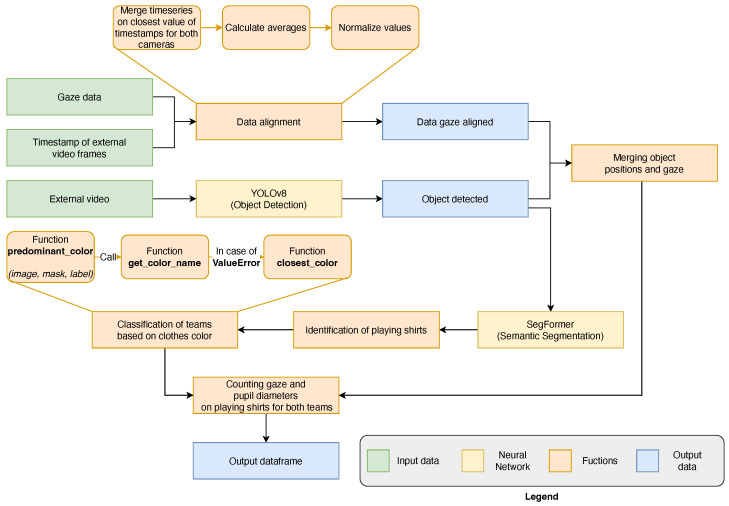
Overview of the system including the function depicted in Figure 2 and Figure 6.

After semantic segmentation, the color of the clothes extracted are preprocessed in order to make the final classification easer: first, the binary mask of the SegFormer is extracted and then, the coordinates of the Figure 5a are used to crop the original image, as shown in Figure 5b. Finally, the Figure 5a are multiplied with Figure 5b to delete the noise around the clothes resulting in Figure 5c. The final step reported in Figure 6 is color detection from clothing to facilitate team assignment. Once an individual is detected by YOLOv8N, their clothing segmented by SegFormer, and the user’s gaze is confirmed on the apparel, its color is identified to categorize the associated data with one of the two teams. The color of this shirt is identified with the Algorithms A1 and A2, and in case the color does not match with one of the known colors, the Algorithm A3 is run. Subsequently the results, including gaze data with the color found and pupil diameter statistics, are saved. The diagram in Figure 7 shows the general operation of the system presented in this work. Considering the computational power of modern laptop and the frame rate of Neon Pupil Labs external camera, the pipeline is capable of real-time operation, allowing for gaze position and pupillometry inference for each frame and output using YOLOv8N and SegFormer neural networks sequentially to obtain immediate results. Future studies will also include automatic semantic sport scene classification to perform the event detection and automatize the whole system for real-time usage [47,48,49].

Following data acquisition, expert annotators label the video recordings to indicate when Team A and Team B were in offensive or defensive positions. This labeling is performed for both coach wearers (Team A, Team B) and referee wearers (lead, tail). Subsequently, the features is extracted for each of the four observers: the number of gazes and the pupil diameter when observing Team A or Team B in both offensive and defensive phases.

## 3. Experimental Results

The system presented in this study is designed and optimized for basketball, but it is principally applicable to all team sports that meet the following criteria: (1) opposing teams must wear uniforms with distinctly different colors, preferably a light and a dark color; (2) the distance between the eye tracker device wearer and the observes subject should not exceed 15 m, as this represents the operational range for which the system was tested and validated (i.e., the distance between the the tail referee and the basketball hoop). The following paragraph will illustrate an example of the system’s application during a basketball game.

### 3.1. Preliminary Findings

Expert evaluators first verify the system’s accuracy in correctly classifying the players’ jerseys based on color, confirming its ability to reliably distinguish between Team A (light color uniforms) and Team B (dark color uniforms) within the specified operational distance. Following this validation, a preliminary analysis of the extracted features is conducted. Despite the limited number of samples collected in this initial phase, the results suggest potentially significant differences in both pupil diameter and gaze patterns among the different observers (lead referee, tail referee, Coach Team A, Coach Team B) across various game phases (offensive and defensive actions, both while looking Team A and Team B). Specifically, initial observations indicate variations in visual attention allocation (reflected in the number and duration of gazes on each team) and physiological arousal levels (inferred from pupil diameter changes) depending on the observer’s role (e.g., referee vs. coach) and the specific context of the game (e.g., observing one’s own team on offense vs. defense).

The analysis and related outcomes of the features extracted from the system and described in Section 2.3, namely gaze count and mean pupil diameter associated with the observation of Teams A and B by both coach and referee, are described below.

### 3.2. Preliminary Statistical Analyses

In this study, a preliminary statistical analysis is conducted on two variables: the number of total gazes and pupil diameter, related to the referees (both tail and lead) and the coaches of both teams, across attacking and defensive situations. On the data collected from the referees, a *t*-tests are implemented to compare gazes and pupil diameter between the tail and lead referees observing the same team. For the data collected from the coaches, the analysis of gazes and pupil diameter is performed while they observes either their own team or the opposing team in both situations (attack and defense). Subsequently, for both coaches, a 2 × 2 ANOVA is performed on gazes as a within-subject factor during gameplay (either attack or defense) and the two coaches as between-subject factor. An additional 2 × 2 ANOVA is performed, considering pupil diameter during game action (whether attack or defense) with the same factors as the previous. The next paragraphs will describe the results. For all analysis, a value of α=0.05 is set as significance threshold.

### 3.3. Referee

Table 1 compares pupil diameter and gaze count for both referees (trail and lead). For Team B, the mean pupil diameter of the trail referee is lower than that of the lead referee, and this difference is statistically significant ( p<0.001 ). In contrast, for Team A, the *p*-value for pupil diameter is at the threshold of statistical significance ( p=0.050 ), suggesting a possible trend, though it is less conclusive than for Team B. Regarding gaze counts, no statistically significant differences were observes between trail and lead referees for either Team A or Team B. Similarly, the overall “Total” rows show no statistically significant differences in either pupil diameter or gaze count (the sum of GAZE Team A and GAZE Team B for both referee roles) when comparing trail and lead referees across both teams combined.

### 3.4. Coach Team A

In Table 2, preliminary findings on the Coach Team A’s visual behavior are presented, comparing pupil diameter and gaze count during offensive (“Attack”—own team in attack) and defensive (“Defense”-oppositive team in defense) game phases. For pupil diameter no significant difference was observes either when the coach was observing the own team or the opposing team during both offensive and defensive phases. However, a statistically significant difference emerges for gaze count Team A ( p=0.032 ), indicating that Coach Team A allocates notably more visual attention to their own players during offensive plays, potentially reflecting a strategic focus on attack. While gaze count for Coach Team A looking at Team B shows a trend of higher gazes during the attack phase of the oppositive team, it is not statistically significant. The total gaze count (the sum of GAZE Team A and GAZE Team B for both conditions, “Attack” and “Defense”) displays a statistically significant difference ( p=0.029 ), strongly indicating that the Coach Team A exhibits a higher total number of gazes across both teams during offensive plays, suggesting a high cognitive demanding role during these periods. It is important to emphasize that these preliminary data, in order to be verified, require more data under different conditions in order to eliminate possible bias.

### 3.5. Coach Team B

In Table 3, preliminary findings regarding the visual behavior of the Coach Team B are presented, comparing pupil diameter during offensive (“Attack”-own team in defense) and defensive (“Defense”–oppositive team in attack) game phases. Values in bold indicate statistically significant differences. An interesting result is observes for pupil diameter of Coach Team B when looking at the own team in defense: the pupil diameter is significantly higher during the defensive phase than during the offensive phase ( p=0.016 ). This suggests that the Coach Team B experiences a greater cognitive load when their team is on defense, possibly due to the need to closely monitor opposing actions and anticipate threats or due to its position on the game field. Regarding gaze count by the Coach Team B on Team A, the number of gazes on the opponent players is significantly higher during the defensive phase as compared to the attack ( p=0.046 ). This indicates that the Coach Team B pays more visual attention to the opposing team when the own team is in the defensive phase. Gaze count by Coach Team B on the own team also shows a significant difference ( p=0.050) , with a higher number of gazes on their own players during defense as compared to attack. This could reflect the coach’s need to closely monitor the own players’ positioning and reactions during defense. At an aggregated level, pupil diameter total is significantly higher in the defensive phase than in the attack phase ( p=0.001 ). These overall results suggest that the defensive phase increase the cognitive load on the coach. Finally, total gazes (the sum of GAZE Team A and GAZE Team B for both conditions, “Attack” and “Defense”) are also significantly higher during defense as compared to the attack phase ( p=0.047 ). This indicates that the Coach Team B makes a significantly greater total number of gazes during defensive phases, suggesting more attention in these situations.

### 3.6. ANOVA Study Design

Table 4 presents the mean gaze counts for coaches of Team A and Team B across offensive and defensive game phases, revealing distinct visual strategies. An ANOVA 2 × 2 (within-subjects factor: game phase; between-subjects factor: coach) shows a significant main or interaction effect ( $F_{\left(1,14\right)}=7.505$ ; p=0.016 ), indicating that observes differences in gaze patterns are not random. Specifically, the Coach Team A exhibits substantially higher gaze counts during offensive plays compared to defensive plays. On the contrary, the Coach Team B demonstrated a markedly higher number of gazes during defensive plays versus offensive phase. This suggests different cognitive patterns enacted during the game but further data is needed to verify the individual coaching difference of the two coaches.

Table 5 presents the mean pupil diameter for coaches of Team A and Team B during offensive and defensive game phases. A second ANOVA 2 × 2 design is used, with “game phase” as a within-subjects factor and “coach” as a between-subjects factor. The ANOVA results ( $F_{\left(1,14\right)}=4.678$ ; p=0.048 ) indicate a statistically significant effect, suggesting variations in cognitive load. Specifically, the data show a greater mean pupil diameter when a coach’s team is playing in their own half of the game field. For the Coach Team A, pupil diameter is slightly higher during attack compared to defense. Given that Team A is typically in their own defensive half during defensive plays, this aligns with the interpretation of increased diameter when playing in their own half. Conversely, the Coach Team B exhibits a higher pupil diameter during defense compared to attack. As Team B is in their own defensive half during defensive plays, this finding strongly supports the notion of increased cognitive load when their team is operating in their own half of the court. This suggests that the defensive phase, require a higher cognitive demand.

While these findings are indicative and highlight the system’s potential to capture subtle behavioral and physiological differences, further data collection across multiple games and observers, followed by rigorous statistical analysis, is necessary to confirm these trends and draw definitive conclusions about the visual strategies and cognitive load of referees and coaches in basketball.

## 4. Discussion

In this work, a novel CV system is presented, which is designed to analyze the eye movements and pupillometry data of observers (referees and coaches) during a live team sport game, specifically basketball. The system integrates data from a wearable eye-tracker (Pupil Lab Neon^®^) which has an internal and an external camera, with two pre-trained AI models: a CNN (YOLOv8N) for player detection and tracking, and a transformer (SegFormer) for semantic segmentation of clothing. This combination allows the system to identify players within the observer’s POV, segment their uniforms, assign each uniform to its respective team based on color, and correlate this information with the observer’s gaze point and pupil diameter on a frame-by-frame basis. By synchronizing these data streams and incorporating manual annotations by experts of game events (offensive/defensive phases), the system extracts features such as the number of gazes and average pupil diameter associated with observing specific teams during different game actions. The preliminary results, although based on limited data, suggest the system’s capability to reveal differences in visual attention patterns and potential physiological arousal between different roles (referees vs. coaches) and game situations.

Specifically, our preliminary findings highlight the system’s potential to detect distinct visual attention patterns based on the referee’s position on the field, particularly when observing the team in defense compared to the team in attack, suggesting a higher cognitive load during the evaluation of defensive situations and movements. Regarding the data recorded from the coaches as well, our system appears to effectively detect the variables of interest, highlighting statistically significant differences in cognitive load (pupil diameter) and visual attention (gaze count).

The use of neural networks for real-time object detection allows us to overcome the limitation of static AOI by dynamically defining them according to the attentional focus of the observer in the game context. This approach is particularly promising for analyses in complex, ecological environments such as team sports.

Despite these promising initial steps, several areas warrant further development and consideration. Currently, the system relies on manual labeling of game events. Future iterations should incorporate automatic scene classification techniques specific to sports [47,48,49,50,51] to fully automate the analysis pipeline, making it more scalable and suitable for real-time applications. Furthermore, the broader adoption and comparison of findings from eye-tracking studies in sports (and other fields) are hampered by the lack of standardized data formats that make these systems tool-dependent. A crucial future direction for the field is the development and adoption of a common standard for storing and sharing eye-tracking data and metadata, analogous to established formats like the Brain Imaging Data Structure (BIDS) for neuroimaging data [52,53], the European Data Format (EDF) for clinical time-series recordings [54], or the Digital Imaging and Communications in Medicine (DICOM) for medical imaging [55,56,57]. Such standardization would greatly enhance data reproducibility, software development [58,59,60,61,61] facilitate meta-analyses, and foster collaboration.

Future technical developments of the system presented here will focus on implementing more sophisticated, ad-hoc features specifically tailored for eye-tracking data analysis in dynamic environments. This includes exploring advanced data fusion techniques, building upon the initial integration of gaze, pupillometry, and AI-driven video analysis demonstrated in this work. The potential for real-time operation is a key advantage of the chosen lightweight neural networks. Future versions aim to leverage this capability to build online feedback systems that can instantaneously identify where an observer is looking and classify what they are observing. Integrating this eye-tracking analysis system with other portable sensor technologies, such as hand-tracking devices (e.g., Leap Motion [14] or EEG devices [15,16]), could provide a more holistic understanding of observer behavior, combining visual attention with motor actions or gestural communication. Such multi-modal systems hold significant potential for applications in sports science, including detailed performance analysis [33], identification and mitigation of perceptual-cognitive biases, optimization of training protocols, and potentially contributing to risk reduction strategies [32].

While the proposed system demonstrates promising capabilities for real-time analysis of gaze and pupillometric data in ecological sports contexts, certain limitations warrant careful consideration. Firstly, the accuracy of the eye-tracking device (Pupil Labs Neon), although sufficient for general attentional mapping (∼1.3° angular error with offset correction), may be insufficient for fine-grained gaze localization in fast-paced visual environments such as basketball. Minor calibration drifts or head-mounted motion artifacts can introduce noise in gaze estimation, especially during dynamic gameplay, potentially affecting the spatial precision of gaze-to-object mapping. Secondly, environmental factors, including varying lighting conditions (e.g., glare from court lights), occlusions (e.g., overlapping players), and background visual clutter, may influence both the performance of the DL models (e.g., YOLOv8N, SegFormer) and the reliability of pupillometric measures, such as pupil dilation, which is known to be light-sensitive. Additionally, the current study is based on a limited dataset collected, which restricts generalizability. Future research should aim to systematically quantify these influences through controlled comparisons, incorporate automated recalibration routines, and test the robustness of the system under different environmental and gameplay conditions. These improvements will enhance system reliability and ecological validity, paving the way for more scalable applications in sport performance monitoring and cognitive workload assessment.

## 5. Conclusions

This study introduces an AI-powered framework for analyzing eye-tracking data in the complex, ecological setting of a live basketball game. It represents a step towards leveraging advanced AI and wearable technology to gain deeper insights into the cognitive processes, decision-making, and visual strategies employed by key personnel like referees and coaches in high-pressure sporting environments. Continued development, validation with larger datasets, develop the real-time application and integration with emerging standards and technologies will be crucial to realizing the full potential of this approach.

## Figures and Tables

**Table 1:** Comparison of pupil diameter (DIA) and gaze count (GAZE) for Lead and Tail Referees Across Teams (A and B). In bold, the significative results of the analysis. DIA Team A: The referee’s (Tail or Lead) pupil diameter when looking at team A; DIA Team B: The referee’s (Tail or Lead) pupil diameter when looking at team B; DIA Total: The referee’s (Tail or Lead) pupil diameter when looking at team A or team B; GAZE Team A: Number of gazes by the referee (Tail or Lead) when looking at team A; GAZE Team B: Number of gazes by the referee (Tail or Lead) when looking at team B; GAZE Total: Total number of gazes by the referee (Tail or Lead) when looking at team A or team B.

	Tail Referee	Lead Referee	
	Mean ± s.d.	Mean ± s.d.	*p*-Value
DIA Team A	5.386 ± 0.127	5.370 ± 0.0567	**0.050**
GAZE Team A	23.500 ± 10.607	22.67 ± 5.033	0.127
DIA Team B	5.266 ± 0.278	5.404 ± 0.0218	**<0.001**
GAZE Team B	86.50 ± 38.891	87.33 ± 31.533	0.697
DIA total	5.326 ± 0.0757	5.387 ± 0.0379	0.122
GAZE total	110.00 ± 49.497	110.00 ± 26.514	0.176

**Table 2:** Comparison of pupil diameter (DIA) and gaze count (GAZE) During Attack and Defense Phases for the Coach of Team A. Statistically significant results are highlighted in bold. DIA Team A: The Coach Team A pupil diameter when looking at team A; DIA Team B: The Coach Team A pupil diameter when looking at team B; DIA total: The Coach Team A pupil diameter when looking at team A or team B; GAZE Team A: Number of gazes by the The Coach Team A when looking at team A; GAZE Team B: Number of gazes by the The Coach Team A when looking at team B; GAZE total: Number of gazes by the The Coach Team A when looking at team A or team B.

Coach Team A			
	Attack (Mean ± s.d.)	Defense (Mean ± s.d.)	*p*-Value
DIA Team A	3.69 ± 0.138	3.908 ± 0.164	0.702
GAZE Team A	22.17 ± 25.269	7.11 ± 6.373	**0.032**
DIA Team B	3.65 ± 0.160	3.707 ± 0.394	0.321
GAZE Team B	57.58 ± 44.771	33.00 ± 19.660	0.071
DIA total	3.52 ± 0.594	3.590 ± 0.847	0.464
GAZE total	79.75 ± 67.617	40.11 ± 23.730	**0.029**

**Table 3:** Comparison of pupil diameter (DIA) and gaze count (GAZE) for Team B Coach During Attack and Defense Phases of its own team. Statistically significant results are highlighted in bold. DIA Team A: The Coach Team B pupil diameter when looking at team A; DIA Team B: The Coach Team B pupil diameter when looking at team B; DIA total: The Coach Team B pupil diameter when looking at team A or team B; GAZE Team A: Number of gazes by the The Coach Team B when looking at team A; GAZE Team B: Number of gazes by the The Coach Team B when looking at team B; GAZE total: Number of gazes by the The Coach Team B when looking at team A or team B.

Coach Team B			
	Attack (Mean ± s.d.)	Defense (Mean ± s.d.)	*p*-Value
DIA Team A	3.96 ± 0.202	4.203 ± 0.148	0.627
GAZE Team A	3.86 ± 6.040	90.57 ± 120.166	**0.046**
DIA Team B	3.60 ± 0.567	4.249 ± 0.125	**0.016**
GAZE Team B	12.14 ± 6.176	62.43 ± 85.186	**0.050**
DIA total	3.21 ± 1.116	4.226 ± 0.114	**0.001**
GAZE total	16.00 ± 10.312	153.00 ± 204.219	**0.047**

**Table 4:** Mean Gaze Counts (±s.d.) for Coach Team A and Coach Team B during Attack and Defense Phases.

	Coach Team A	Coach Team B
Gazes Attack	100.44 ± 65.73	16.00 ± 10.31
Gazes Defense	40.11 ± 23.72	153.00 ± 204.21
Result	** $F_{\left(1,14\right)}=7.505;\;p=0.016$ **	

**Table 5:** Mean Pupil Diameter (±s.d.) for Coach Team A and Coach Team B during Attack and Defense Phases.

	Coach Team A	Coach Team B
Pupil Diameter Attack	3.66 ± 0.13	3.21 ± 1.11
Pupil Diameter Defense	3.59 ± 0.84	4.22 ± 1.11
Result	** $F_{\left(1,14\right)}=4.678;\;p=0.048$ **	

## Data Availability

The datasets presented in this article are not readily available because the analysis the results is not the scope of the manuscript. Requests to access the datasets should be directed to the Corresponding Author.

## Abbreviations

AI	Artificial Intelligence
AR	Augmented Reality
AOI	Area of Interest
BIDS	Brain Imaging Data Structure
CNN	Convolutional Neural Network
CV	Computer Vision
DICOM	Digital Imaging and Communications in Medicine
DL	Deep Learning
EDF	European Data Format
EEG	Electroencephalography
FOV	Field of View
IMU	Inertial Measurement Unit
IR	InfraRed
ML	Machine Learning
POV	Point of View
SegFormer	Simple and Efficient Design for Semantic Segmentation with Transformers
SVR	Support Vector Regression
QE	Quiet Eye
YOLOv8N	You Only Look Once neural network v. 8N

## Appendix A. Algorithms

### Appendix A.1. Nearest Color

**Algorithm A1** Find the nearest color
1: **function** closest_color( requested_color ) 2: **for** (key,name)∈webcolors.CSS3_HEX_TO_NAMES.items() **do** 3: (r_c,g_c,b_c)←webcolors.hex_to_rgb(key) 4: $distance\leftarrow {\left(r\_c-requested\_color\left[0\right]\right)}^{2}+{\left(g\_c-requested\_color\left[1\right]\right)}^{2}+{\left(b\_c-requested\_color\left[2\right]\right)}^{2}$ 5: min_colors[distance]←name 6: **end for** 7: **return** min_colors[min(min_colors.keys())] 8: **end function**

### Appendix A.2. Name of an RGB Color

**Algorithm A2** Get the name of an RGB color
1: **function** get_color_name( rgb_color ) 2: **try** 3: **return** webcolors.rgb_to_name(rgb_color) 4: **catch** *ValueError* 5: **return** closest_color( rgb_color ) 6: **end function**

### Appendix A.3. Predominant Color

**Algorithm A3** Calculates the predominant color in a masked region
1: **function** predominant_color( image , mask , label_to_segment ) 2: **if** label_to_segment∉mask **then** 3: **raise** ValueError 4: **end if** 5: mask_segment←(mask==label_to_segment) 6: masked_image←image[mask_segment] 7: masked_image_reduced←(masked_image//32)∗32 8: (colors,count)←np.unique(masked_image_reduced.reshape(−1,3),axis=0,return_counts=True) 9: predominant_color_rgb←colors[count.argmax()] 10: **return** get_color_name( tuple(predominant_color_rgb) ) 11: **end function**

## References

[1] Cullipher S., Hansen S.J., VandenPlas J.R. (2018). Eye tracking as a research tool: An introduction. Eye Tracking for the Chemistry Education Researcher.

[2] Kanat E.A., Şimşek D. (2021). The ‘Quiet Eye’ and motor performance in basketball free throw shooting. Phys. Educ. Stud..

[3] Valliappan N., Dai N., Steinberg E., He J., Rogers K., Ramachandran V., Xu P., Shojaeizadeh M., Guo L., Kohlhoff K. (2020). Accelerating eye movement research via accurate and affordable smartphone eye tracking. Nat. Commun..

[4] Brunyé T.T., Drew T., Weaver D.L., Elmore J.G. (2019). A review of eye tracking for understanding and improving diagnostic interpretation. Cogn. Res. Princ. Implic..

[5] Giancamilli F., Galli F., Chirico A., Fegatelli D., Mallia L., Palombi T., Lucidi F. (2022). High-pressure game conditions affect quiet eye depending on the player’s expertise: Evidence from the basketball three-point shot. Brain Sci..

[6] Moeinirad S., Abdoli B., Farsi A., Ahmadi N. (2022). Training visual attention improves basketball three-point shot performance under pressure. Sport Sci. Health.

[7] Ruiz A.J., Albaladejo C., Reina R., Moreno F.J. (2023). Basketball referee’s gaze behavior and stimulus selection in relation to visual angle perspective and officiating mechanics and expertise. Eur. J. Hum. Mov..

[8] Klatt S., Noël B., Nicklas A., Schul K., Seifriz F., Schwarting A., Fasold F. (2021). Gaze behavior and positioning of referee teams during three-point shots in basketball. Appl. Sci..

[9] Ben Chikha H., Zoudji B., Khacharem A. (2024). The role of coach’s gaze guidance on memorization of tactical movements in basketball: An eye tracking study. Ger. J. Exerc. Sport Res..

[10] Rui M., Fernando M., Ricardo G., Martinho D.V., Rui M., Moore S.A., Coelho-e Silva M.J., Gonçalo D. (2023). Visual Information in Basketball Jump-Shots: Differences between Youth and Adult Athletes. J. Hum. Kinet..

[11] Ueyama Y., Harada M. (2024). Basketball free-throw training with augmented reality-based optimal shot trajectory for novice shooters. Sci. Rep..

[12] Chikha H.B., Zoudji B., Khacharem A. (2024). Coaches’ pointing gestures as means to convey tactical information in basketball: An eye-tracking study. Int. J. Sport Exerc. Psychol..

[13] Piras A. (2025). The role of the peripheral target in stimulating eye movements. Psychol. Sport Exerc..

[14] Polsinelli M., Matteo A.D., Lozzi D., Mattei E., Mignosi F., Nazzicone L., Stornelli V., Placidi G. (2024). Portable Head-Mounted System for Mobile Forearm Tracking. Sensors.

[15] Thompson T., Steffert T., Ros T., Leach J., Gruzelier J. (2008). EEG applications for sport and performance. Methods.

[16] Sultanov M.B., İsmailova K.Y. (2020). Wireless EEG system for Sport Science: Quantitative analysis of movement. Proceedings of the 2020 7th International Conference on Behavioural and Social Computing (BESC).

[17] Alemanno M., Di Pompeo I., Marcaccio M., Canini D., Curcio G., Migliore S. (2025). From Gaze to Game: A Systematic Review of Eye-Tracking Applications in Basketball. Brain Sci..

[18] Zhao C., Liu N., Li S., Zhao X. (2024). Investigation of eye movement characteristics during free throws at varying intensities among basketball players and its correlation with free throw percentage. PLoS ONE.

[19] Meyer J., Fasold F., Schul K., Sonnenschein M., Klatt S. (2022). The defender’s vision—Gaze behavior of one-on-one defenders in basketball. J. Sport Exerc. Psychol..

[20] Tatara S., Toda H., Maeda F., Ito A., Handa T. (2024). Comparison of the Saccadic eye movement ability of female professional basketball players and non-athletes. Appl. Sci..

[21] Placidi G., Cinque L., Foresti G.L., Galassi F., Mignosi F., Nappi M., Polsinelli M. (2025). A Context-Dependent CNN-Based Framework for Multiple Sclerosis Segmentation in MRI. Int. J. Neural Syst..

[22] Ma J., He Y., Li F., Han L., You C., Wang B. (2024). Segment anything in medical images. Nat. Commun..

[23] Antonelli M.G., Beomonte Zobel P., Manes C., Mattei E., Stampone N. (2024). Emotional intelligence for the decision-making process of trajectories in collaborative robotics. Machines.

[24] Jan Z., Ahamed F., Mayer W., Patel N., Grossmann G., Stumptner M., Kuusk A. (2023). Artificial intelligence for industry 4.0: Systematic review of applications, challenges, and opportunities. Expert Syst. Appl..

[25] Goud P.S.H.V., Roopa Y.M., Padmaja B. (2019). Player performance analysis in sports: With fusion of machine learning and wearable technology. Proceedings of the 2019 3rd International Conference on Computing Methodologies and Communication (ICCMC).

[26] Mgaya G.B., Liu H., Zhang B. (2021). A survey on applications of modern deep learning techniques in team sports analytics. Proceedings of the 12th International Conference on Soft Computing and Pattern Recognition (SoCPaR 2020) 12.

[27] Lakshmi P.Y., Sanjaykumar S., Dharuman M., Elangovan A. (2024). Using Support Vector Regression Kernel Models for Cricket Performance Prediction in the Womens Premier League 2024. Phys. Educ. Theory Methodol..

[28] Tian C., De Silva V., Caine M., Swanson S. (2019). Use of machine learning to automate the identification of basketball strategies using whole team player tracking data. Appl. Sci..

[29] Du D., Chai L., Li M., Jiang L., Choosakul C., Liu S. (2024). Extracting Features from Foul Actions of Basketball Players in Real Time Using Machine Vision. Int. J. Comput. Intell. Syst..

[30] Penumala R., Sivagami M., Srinivasan S. (2019). Automated goal score detection in football match using key moments. Procedia Comput. Sci..

[31] Zhang Q., Yu L., Yan W. (2025). AI-Driven Image Recognition System for Automated Offside and Foul Detection in Football Matches Using Computer Vision. Int. J. Adv. Comput. Sci. Appl..

[32] Zhu D., Zhang H., Sun Y., Qi H. (2021). Injury risk prediction of aerobics athletes based on big data and computer vision. Sci. Program..

[33] Rajšp A., Fister I. (2020). A systematic literature review of intelligent data analysis methods for smart sport training. Appl. Sci..

[34] Amankwah G., Morgan-Darko W., Yang C. (2019). Real-time analysis and profiling of coordinated movements in two-dimensional space using footage from multiple cameras. Int. J. Comput. Appl..

[35] Parker S., Duthie G., Robertson S. (2024). A framework for player movement analysis in team sports. Front. Sport. Act. Living.

[36] Torres-Ronda L., Beanland E., Whitehead S., Sweeting A., Clubb J. (2022). Tracking systems in team sports: A narrative review of applications of the data and sport specific analysis. Sport. Med.-Open.

[37] Hammes F., Hagg A., Asteroth A., Link D. (2022). Artificial intelligence in elite sports—A narrative review of success stories and challenges. Front. Sport. Act. Living.

[38] Naraine M., Wanless L. (2020). Going all in on ai. Sport. Innov. J..

[39] Li B., Xu X. (2021). Application of artificial intelligence in basketball sport. J. Educ. Health Sport.

[40] Naik B.T., Hashmi M.F., Bokde N.D. (2022). A comprehensive review of computer vision in sports: Open issues, future trends and research directions. Appl. Sci..

[41] Redmon J., Divvala S., Girshick R., Farhadi A. You only look once: Unified, real-time object detection. Proceedings of the IEEE Conference on Computer Vision and Pattern Recognition.

[42] Xie E., Wang W., Yu Z., Anandkumar A., Alvarez J.M., Luo P. (2021). SegFormer: Simple and efficient design for semantic segmentation with transformers. Adv. Neural Inf. Process. Syst..

[43] Paszke A., Gross S., Chintala S., Chanan G., Yang E., DeVito Z., Lin Z., Desmaison A., Antiga L., Lerer A. Automatic differentiation in PyTorch. Proceedings of the NIPS-W.

[44] Harris C.R., Millman K.J., Van Der Walt S.J., Gommers R., Virtanen P., Cournapeau D., Wieser E., Taylor J., Berg S., Smith N.J. (2020). Array programming with NumPy. Nature.

[45] Hunter J.D. (2007). Matplotlib: A 2D graphics environment. Comput. Sci. Eng..

[46] Pedregosa F., Varoquaux G., Gramfort A., Michel V., Thirion B., Grisel O., Blondel M., Prettenhofer P., Weiss R., Dubourg V. (2011). Scikit-learn: Machine Learning in Python. J. Mach. Learn. Res..

[47] Assfalg J., Bertini M., Colombo C., Del Bimbo A., Nunziati W. (2003). Semantic annotation of soccer videos: Automatic highlights identification. Comput. Vis. Image Underst..

[48] Huang C.L., Shih H.C., Chao C.Y. (2006). Semantic analysis of soccer video using dynamic Bayesian network. IEEE Trans. Multimed..

[49] Afzal M., Shah J.H., ur Rehman S., Khokhar F.A., Yasmin M., Kadry S. (2024). Automated soccer event detection and highlight generation for short and long views. Multimed. Tools Appl..

[50] Choroś K. (2009). Video shot selection and content-based scene detection for automatic classification of TV sports news. Internet–Technical Development and Applications.

[51] Choroś K., Pawlaczyk P. (2010). Content-based scene detection and analysis method for automatic classification of TV sports news. Rough Sets and Current Trends in Computing: Proceedings of the 7th International Conference, RSCTC 2010, Warsaw, Poland, 28–30 June 2010.

[52] Gorgolewski K.J., Auer T., Calhoun V.D., Craddock R.C., Das S., Duff E.P., Flandin G., Ghosh S.S., Glatard T., Halchenko Y.O. (2016). The brain imaging data structure, a format for organizing and describing outputs of neuroimaging experiments. Sci. Data.

[53] Pernet C.R., Appelhoff S., Gorgolewski K.J., Flandin G., Phillips C., Delorme A., Oostenveld R. (2019). EEG-BIDS, an extension to the brain imaging data structure for electroencephalography. Sci. Data.

[54] Kemp B., Värri A., Rosa A.C., Nielsen K.D., Gade J. (1992). A simple format for exchange of digitized polygraphic recordings. Electroencephalogr. Clin. Neurophysiol..

[55] Mildenberger P., Eichelberg M., Martin E. (2002). Introduction to the DICOM standard. Eur. Radiol..

[56] Halford J.J., Clunie D.A., Brinkmann B.H., Krefting D., Rémi J., Rosenow F., Husain A., Fürbass F., Ehrenberg J.A., Winkler S. (2021). Standardization of neurophysiology signal data into the DICOM^®^ standard. Clin. Neurophysiol..

[57] Matteo A.D., Lozzi D., Mignosi F., Polsinelli M., Placidi G. (2025). A DICOM-based Standard for Quantitative Physical Rehabilitation. Comput. Struct. Biotechnol..

[58] Lozzi D., Di Pompeo I., Marcaccio M., Ademaj M., Migliore S., Curcio G. (2025). SPEED: A Graphical User Interface Software for Processing Eye Tracking Data. NeuroSci.

[59] Sogo H. (2013). GazeParser: An open-source and multiplatform library for low-cost eye tracking and analysis. Behav. Res. Methods.

[60] Filetti M., Tavakoli H.R., Ravaja N., Jacucci G. (2019). PeyeDF: An eye-tracking application for reading and self-indexing research. arXiv.

[61] Lejarraga T., Schulte-Mecklenbeck M., Smedema D. (2016). The pyetribe: Simultaneous eyetracking for economic games. Behav. Res. Methods.

